# Prize Offered to the Medical Students of the United States

**Published:** 1860-05

**Authors:** 


PRIZE OFFERED TO THE MEDICAL STUDENTS OF THE UNITED STATES.—Dr. John O'Reilly, of New York, will award a prize of two hundred and fifty dollars for the best essay on "The Anatomy and Physiology of the Animal and Organic Nervous Systems." The essays must be sent to Dr. O'Reilly by the 1st of March, 1861, and the successful competitor must declare on his honor that he has not been assisted in his production by any medical man. The same prize is offered for the best paper on the same subject, the essays to be handed in on or before the 1st of March, 1862.

